# Improved quality control of [^177^Lu]Lu-PSMA I&T

**DOI:** 10.1186/s41181-023-00191-6

**Published:** 2023-03-27

**Authors:** Martin Kraihammer, Piotr Garnuszek, Andreas Bauman, Michael Maurin, Manuel Alejandre Lafont, Roland Haubner, Elisabeth von Guggenberg, Michael Gabriel, Clemens Decristoforo

**Affiliations:** 1 Department of Nuclear Medicine, Medical University Innsbruck, Anichstr. 35, 6020 Innsbruck, Austria; 2 Department of Nuclear Medicine and Endocrinology, Kepler University Hospital, Linz, Austria; 3 Radioisotope Centre POLATOM, National Centre for Nuclear Research, Otwock, Poland; 4 Division of Radiopharmaceutical Chemistry, University Hospital Basel, Basel, Switzerland

**Keywords:** PSMA, [^177^Lu]Lu-PSMA I&T, Zadavotide guraxetan, Radionuclide therapy, Quality control, HPLC, TLC, Validation

## Abstract

**Background:**

Targeted radionuclide therapy with [^177^Lu]Lu-PSMA I&T (zadavotide guraxetan) has proven high efficacy and safety in treating patients with advanced prostate cancer worldwide. Several methods to determine the radiochemical purity have been reported but also limitations in the HPLC analysis due to retention of the sample and tailing effects when using standard gradients containing trifluoroacetic acid (TFA). We here report on the validation of a method for quality control of [^177^Lu]Lu-PSMA I&T including determination of radiochemical purity, identity testing and limit test for PSMA I&T by HPLC using a Phosphate buffer /Acetonitrile gradient system, complemented with a TLC system with 0.1N Citrate buffer pH 5 as mobile phase including validation of the methods, batch and stability data as well as identification of the main radiochemical impurity by mass spectrometry.

**Results:**

The described HPLC method met the defined acceptance criteria in terms of accuracy, specificity, robustness, linearity, range and LOQ. HPLC analysis revealed symmetrical peaks and quantitative recovery from the column. Batch data showed a radiochemical purity > 95% as determined by HPLC, stability data a pronounced degradation due to radiolysis, which could be limited by addition of ascorbic acid, dilution and storage at low temperatures. The main radiochemical impurity was found to be the de-iodinated form of [^177^Lu]Lu-PSMA I&T. TLC analysis allowed to determine the amount of free Lu-177 even in the presence of DTPA in the final formulation.

**Conclusion:**

Overall the described combination of HPLC and TLC provides a reliable tool for quality control of [^177^Lu]Lu-PSMA I&T.

**Supplementary Information:**

The online version contains supplementary material available at 10.1186/s41181-023-00191-6.

## Introduction

Targeting the prostate specific membrane antigen (PSMA) has gained an enormous impact on Nuclear Medicine patient care starting with the clinical success of PET imaging with [^68^Ga]Ga-PSMA-11 ten years ago (Afshar-Oromieh et al. 2013). This was complemented by the successful implementation of therapeutic application using the Lu-177 labelled PSMA ligands PSMA-617 (vipivotide tetraxetan) (Afshar-Oromieh et al. 2015) and PSMA I&T (zadavotide guraxetan) (Weineisen et al. 2015). [^177^Lu]Lu-PSMA-617 has recently gained approval by the FDA (Pluvicto®), but also [^177^Lu]Lu-PSMA I&T has proven high efficacy and safety in treating patients with advanced prostate cancer worldwide (Bu et al. 2022; John et al. 2022; Heck et al. 2019; Hartrampf et al. 2022), with very comparable outcomes (Schuchardt et al. 2022; Hartrampf et al. 2022). Today [^177^Lu]Lu-PSMA I&T is widely used mainly because of its current better availability for local, in house preparation (Notni 2021). However, so far, no formal quality standards are available. A variety of methods have been reported to determine the radiochemical purity of [^177^Lu]Lu-PSMA I&T. These methods are based on reversed-phase high-performance liquid chromatography (RP-HPLC) employing standard conditions with C-18 columns and acetonitrile (ACN) /water mixtures containing 0.1% Trifluoroacetic acid (TFA) as counterion (Orhon et al. 2022; Weineisen et al. 2014; Iorio et al. 2022; Hooijman et al. 2022; Vyas et al. 2022). Recently, Aalbersberg et al. (2022) reported unspecific retention of [^177^Lu]Lu-PSMA I&T on RP-C-18 HPLC columns using TFA containing solvents, that could be overcome by spiking quality control samples with PSMA I&T precursor. We observed similar phenomena during the analytical development in our laboratory when using TFA-containing RP gradients resulting in low reproducibility and peak tailing. Additionally, HPLC-analysis should not only enable determination of the radiochemical purity but also a proof of identity and a limit test of PSMA I&T and related substances in the preparation. However, the latter is not possible when sample spiking is performed. We report here on a modified HPLC analysis for the quality control of [^177^Lu]Lu-PSMA I&T overcoming the limitations of TFA based methods in terms of recovery and tailing and allowing a limit test for PSMA I&T in radiopharmaceutical preparations, and, at the same time, improving the specificity of the analysis. We provide identification of the main radiochemical impurity, including validation data according to current standards (Gillings et al. 2020) and batch- as well as stability data.

## Materials and methods

If not otherwise indicated, reagents were obtained from VWR International GmbH (Vienna, Austria) or Merck KGaA (Darmstadt, Germany) and were used without further purification.

PSMA I&T was obtained from piCHEM (Raaba-Grambach, Austria).

[^177^Lu]LuCl_3_ was obtained in n.c.a. quality (EndolucinBeta®, ITM Medical Isotopes GmbH, Garching, Germany).

Inactive reference compound ^nat^Lu-PSMA I&T was prepared by reacting 500 µg PSMA I&T with 50 µL Lu-Chloride solution (10 mg LuCl_3_/mL 0.1 N HCl) and 50 µL sodium acetate solution (310 mg sodium acetate trihydrate in 2 mL water) at 90°C for 15 min. The resulting solution was purified over a Sep-Pak C18 Plus Light cartridge (Waters, Vienna, Austria), washed with 5 mL water and eluted with 1 mL 50% Ethanol. The resulting stock solution was stored at -20 °C, ^nat^Lu-PSMA I&T was analysed for purity by HPLC (Method A) and for identity by mass spectrometry.

*Radioactive test solutions*: Test solutions were taken from routine clinical batches, unless otherwise stated. [^177^Lu]Lu-PSMA I&T was prepared using an automated cassette based synthesis module (PharmTracer, Modular Lab, Eckert&Ziegler, Berlin, Germany) as described previously (Petrik et al. 2011) with some modifications. Briefly PSMAI&T (130 µg in 50% water/ethanol per patient with a concentration of 1 mg/mL) were reacted with [^177^Lu]LuCl_3_ (80–100 GBq/µmol PSMA I&T) in ascorbate buffer (Polatom; 50 mg kit ASC-01 dissolved in 1–2 mL of water for injection (10 mL ampoules Fresenius Kabi, Graz, Austria) depending on the activity amount used) at 90 °C for 16.7 min. The crude product was diluted with 2 mL of physiological saline and loaded onto a C18 column for purification. The product was eluted with approx. 2 mL of 50% ethanol in water, diluted with 15 mL of 0.9% NaCl, and sterile filtered by passing the solution through a 0.22μm filter into the product vial containing DTPA (Ditripentat-Heyl®, Berlin, Germany; diluted to 3 mg/mL and added at 20 molar excess over PSMA I&T) and ascorbic acid (Vitamin C-Injektopas®, Pascoe Pharmazeutische Praeparate GmbH, Enzersdorf, Austria; equivalent to 10 mg/GBq).

### HPLC

RP-HPLC analysis was carried out using the following instrumentation (Thermo Fisher Scientific, Vienna, Austria): UltiMate 3000 RS UHPLC pump, UltiMate 3000 auto sampler, UltiMate 3000 column compartment (25 °C oven temperature), UltiMate 3000 Variable Wavelength Detector with UV detection at λ = 200 nm and radio-detector (Gabi Star, Raytest; Straubenhardt, Germany).

The following conditions were applied:

*Method A*: Column: Phenomenex Jupiter 4 µm Proteo 90 Å, 250 × 4.6 mm; Flowrate 1 mL/min; solvent A: 0.03 M Phosphate buffer pH 2.3; solvent B: ACN; gradient: 0–2 min: 23% B, 2–10 min: 23–30% B, 10–12 min: 30–60% B, 12–14 min: 60% B, 14–18 min: 23% B.

*Method B*: Column: Phenomenex Kinetex 5 µm C18 100 Å, 150 × 4.6 mm; Flowrate 1 mL/min; Solvent A: 0.03 M Phosphate buffer pH 2.3; Solvent B: ACN; Gradient: 0–2 min: 20% B, 2–10 min: 20–25% B, 10–12 min: 25–80% B, 12–14 min: 80% B, 14–18 min 20% B.

An injection volume of 10 µL was applied unless otherwise stated.

### Validation of HPLC

For dilution of samples a solvent mixture of 80% HPLC solvent A and 20% solvent B was used. Stock solutions of PSMA I&T and ^nat^Lu-PSMA I&T of 100 µg/mL in the solvent mixture were prepared. Resolution and peak symmetry were calculated according to the European Pharmacopoeia, chapter 2.2.46 Chromatographic separation techniques (Council of Europe, European Pharmacopoeia Commission 2020), correlation coefficient and standard deviations were calculated using Microsoft Excel.

#### Test for Identity

*Accuracy for Identity testing*: The standard deviation of the mean retention time of the peak due to [^177^Lu]Lu-PSMA I&T of 6 independent injections of the test solution was calculated using an acceptance limit of less than 1%.

*Specificity for identity testing*: The retention time of 6 independent injections of the test solution was compared to the UV peak due to ^nat^Lu-PSMA I&T, using a difference in retention time of < 0.5 min as acceptance criteria.

#### Radiochemical purity

*Accuracy and precision for radiochemical purity testing*: The recovery of 6 independent injections of the test solution was calculated as detailed in (Aalbersberg et al. 2022) and (Gillings et al. 2020), using a range of 90–110% as acceptance criteria. It was not possible to derive precision from the variation of RCP values of different injections, as RCP declined over time and with repetition of injections of the same sample.

*Specificity:* A solution of a 1:1 mixture of stock solutions of PSMA I&T and ^nat^Lu-PSMA I&T was prepared (final concentration 50 µg/mL each). Specificity was defined by the resolution of the UV peaks due to both compounds using a resolution of > 2 as acceptance criteria. This test was used both for specificity of radiochemical purity and limit test for PSMA I&T and related substances.

*Linearity and range:* A sample of 300 MBq [^177^Lu]LuCl_3_ (10 µL) was diluted to 1 mL with 20 mg/mL Na/Ca-DTPA mixed with the solvent mixture. Different dilutions in the range of 0.03–300 MBq/mL were injected onto the HPLC system in duplicates (injection volume 5, 10 and 20 µL). The area of the peaks was determined and the injected radioactivity (in Bq) plotted against the area of the generated peaks. Linearity was calculated, using a correlation coefficient of R > 0.99 as acceptance criteria.

Limit of Quantification (LOQ): LOQ was calculated from the peak with a signal/noise ratio > 10. A limit of less than 0.01% of the original solution (300 MBq/mL, injection volume 10 µL) was set as acceptance criteria for the LOQ.

*Robustness:* Robustness was determined varying pH (2.0, 2.5 and 3.0), column temperature (15, 25 and 35 °C), flow rate (0.7, 1.0, 1.3 mL/min) and acetonitrile content (± 2%). The resolution between the peak related to [^177^Lu]Lu-PSMA I&T and the main radiochemical impurity (relative retention time of about 0.6 using the original condition) was calculated using a minimum resolution of 5 as acceptance criteria.

#### Limit test for PSMA I&T and related substances

*Linearity*: The PSMA I&T stock solution (100 µg/mL) was diluted to 10, 5 and 1 µg/mL with the solvent mixture and samples (including the stock solution) injected onto the HPLC system in triplicates. The area of the peak due to PSMA I&T at 200 nm was determined and plotted against the concentration, acceptance criteria was set as R > 0.99.

*Limit of quantification (LOQ)*: LOQ was calculated as the peak area due to PSMA I&T with a signal/noise ratio > 10, acceptance criteria was set to be less than 10 µg/mL.

### Identification of the main radiochemical impurity

100 µL of a 50 µg/mL solution of ^nat^Lu-PSMA I&T in a 1 mL Eppendorf tube was subjected to external irradiation using a ^137^Cs IBL 437L irradiator (CIS US Inc., NY, USA) at a dose rate of about 5 Gy/min for 100 min reaching a dose of 500 Gy. The sample was purified by HPLC (injection volume 50 µL) using HPLC method A and the peaks of the impurity and the main peak due to ^nat^Lu-PSMA I&T were collected and analysed by MALDI-TOF MS.

Mass analysis was conducted on a Bruker microflex® bench-top MALDI-TOF MS (Bruker Daltonics, Bremen, Germany) using dried-droplet method on a micro scout target (MSP96 target ground steel BC, Bruker Daltonics) with α-cyano-4-hydroxycinnamic acid (HCCA, Sigma-Aldrich, Handels GmbH, Vienna, Austria) as matrix. Flex Analysis 2.4 software (Bruker Daltonics, Bremen, Germany) was used for data processing.

### TLC

For TLC two solvent systems were established both based on ITLC-SG strips (Agilent, Vienna, Austria) as solid phase. As mobile Phase 0.1 M Citrate buffer pH 5 (System 1) and ammonium acetate 1 M /methanol 1:1 (System 2) were applied. Validation was performed only for system 1, only specificity parameters are reported herein.

Reference solutions: 3 reference solutions were prepared: [^177^Lu]LuCl_3_ in 0.04 N HCl, [^177^Lu]LuCl_3_ in 0.04 N HCl adjusted to pH 7 with 0.1 N NaOH (representing [^177^Lu]Lu- colloid), [^177^Lu]LuCl_3_ diluted with 50 mg/mL Na-Ca-DTPA ([^177^Lu]Lu-DTPA). Test solution and reference solutions were analysed in triplicates in both systems.

TLC was performed applying 5 µL of a test solution onto ITLC-SG plates developed over a distance of 8 cm and analysed. A Scan-RAM radio-TLC scanner with a PS Plastic/PMT detector (LabLogic Systems, Sheffield, UK) was used to analyse the radiochromatograms.

### Batch data and stability test

The results of 10 routine batches were evaluated, 4 of them using both HPLC methods. Stability of the test solution diluted 1:3 with saline containing 50 mg/mL ascorbic acid was tested over a period of 72 h with storage at RT, 4 °C and -20 °C. Additionally one sample was tested without addition of ascorbic acid and stored at RT.

## Results

### HPLC Method A

**Fig. 1:**
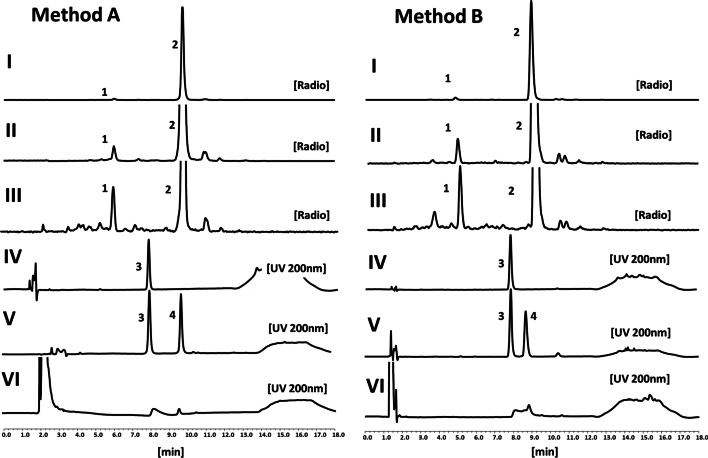
Representative Chromatograms for PSMA I&T. Left: Method A, right: Method B, From top to bottom: I-Radiochromatogram of a test sample of [^177^Lu]Lu-PSMA I&T with a radiochemical purity of about 96%; II- ~ 20 × Zoom of radiochromatogram of test sample of [^177^Lu]Lu-PSMA I&T; III-20 × zoom of radiochromatogram of test sample of [^177^Lu]Lu-PSMA I&T stored at RT for 48 h; IV-UV trace of PSMA I&T standard 200 nm, V-UV trace of a mixture of PSMA I&T and ^nat^Lu-PSMA I&T UV 200 nm; VI- UV-chromatogram of a test sample of [^177^Lu]Lu-PSMA I&T. Peak labels: 1: main radiochemical impurity; 2: [^177^Lu]Lu-PSMA I&T; 3: PSMA I&T; 4: ^nat^Lu-PSMA I&T

Figure 1 shows representative chromatograms of HPLC method A. [^177^Lu]Lu-PSMA I&T elutes with a retention time of about 9.5 min, providing highly symmetrical peaks with peak symmetry of < 1.4. Recovery calculations from 6 consecutive injections resulted in a mean value of 99.0 ± 0.04% indicating that the sample almost quantitatively elutes from the column. The radiochromatograms of test solutions revealed major impurities with relative retention times with respect to [^177^Lu]Lu-PSMA I&T of 0.62, 0.75, 1.12 and 1.21, respectively.

In the UV chromatogram PSMA I&T and ^nat^Lu-PSMA I&T showed retention times of about 8.0 and 9.5 min, respectively. Analysis of the mixture of both compounds provided a mean resolution of 7.12 ± 0.56 (n = 6), showing an excellent separation of PSMA I&T from its lutetium complex and providing the basis for a system suitability test.

Validation data for the HPLC method are summarized in Table 1, indicating that the acceptance criteria were met in all cases. An excellent linearity was found, the range exceeded the acceptance criteria in the upper limit with 600 MBq/mL and the LOQ for the radiometric detection, as well as the LOQ for PSMA I&T with 1 µg/mL were suitable for its intended purpose.

**Table 1:** Validation parameters, acceptance criteria and results for HPLC method A and B

Criteria	Validation parameter	Method	No of repetitions	Aceptance Criteria	Found Method A	Found Method B
Identity	Accuracy	Retention time [^177^Lu]Lu-PSMA I&T	6	sd (%) < 1%	0.57%	0.82%
Identity	Specificity	Retention time [^177^Lu]Lu-PSMA I&T vs ^nat^Lu-PSMA I&T	6	deviation < 0.5 min	0.35 min	0.14 min
Radiochemical purity	(Accuracy, Precision)	Recovery calculation	6	Recovery 90–110%	98.7–101.7%	98.9–102.5%
Radiochemical purity	Specificity	Resolution PSMA I&T vs ^nat^Lu-PSMA I&T	6	Resolution > 3	6.7–7.8	3.7
Radiochemical purity	Robustness	Variation in pH, temperature, flow rate and ACN content	1 per condition, n = 9	Resolution [^177^Lu]Lu PSMA I&T and main impurity > 5	12.0–15.4	12.1–16.7
Radiochemical purity	Linearity	Dilution series [^177^Lu]Lu-DTPA, determine the correlation coefficient	2 per dilution	R > 0.99	0.999	0.999
Radiochemical purity	Range	Dilution series [^177^Lu]Lu-DTPA, determine the concentrations between LOQ und maximum activity	2 per dilution	0.3–300 MBq/mL	0.3–600 MBq/mL	0.3–600 MBq/mL
Radiochemical purity	LOQ	Dilution series [^177^Lu]Lu-DTPA, determine the concentration where peak heigth exceeds 10 × background	2 per dilution	< 0.1% of the initial concentration of the test-solution (300 MBq/mL)	0.05%	0.05%
Limit test for PSMA I&T and related substances	Linearity	Dilution series PSMA I&T	4 per dilution	R > 0.99	0.999	0.999
Limit test for PSMA I&T and related substances	LOQ	Dilution series PSMA I&T, determine the concentration where peak heigth exceeds 10 × background	4 per dilution	< 10 µg/mL	1 µg/mL	1 µg/mL

### HPLC Method B

Figure 1 shows representative chromatograms of HPLC method B. Retention times were similar with 8.9 min, 8.8 min and 8.0 min for [^177^Lu]Lu-PSMA I&T, ^nat^Lu-PSMA I&T and PSMA I&T, respectively. Peak symmetry was slightly better with values of < 1.3, but resolution of PSMA I&T vs. ^nat^Lu-PSMA I&T lower with a mean value of 3.70 ± 0.03, still showing good baseline separation between PSMA I&T and its lutetium complex. Overall validation parameters were very comparable with Method A, details can be found in Table 1. Method B also underwent validation in a different laboratory using a diluted radiolabelled sample with 50 µg/mL PSMA I&T, 25 mg/mL ascorbic acid and ^177^Lu radioactivity concentration of 2.5 GBq/mL (LutaPol, specific activity ca. 500 GBq/mg) and a standard sample containing 125 µg/mL each of PSMA I&T and ^nat^Lu-PSMA I&T mixture in ascorbic buffer. Despite some differences in e.g. retention times, which may be due to equipment differences and the different matrix of test samples, in general the results were comparable. (see Additional file 1: Fig. S1). Retention times were 8.4 min, 8.3 min and 7.6 min for [^177^Lu]Lu-PSMA I&T, ^nat^Lu-PSMA I&T and PSMA I&T, respectively. Peak symmetry was ca. 1.0 and 1.3 for PSMA I&T and ^nat^Lu-PSMA I&T, respectively, and the resolution was 3.1 at 25 µg/mL concentrations for each substance. The radiochromatograms of test solutions revealed major impurities with relative retention times with respect to [^177^Lu]Lu-PSMA I&T of 0.43, 0.71, 1.2 and 1.24, respectively.

### Identification of the main radiochemical impurity

After irradiation with 500 Gy an additional peak was detected on UV in a sample of ^nat^Lu-PSMA I&T using both HPLC method A and B. The peak corresponded in R_t_ to the main radiochemical impurity peak detected in radiochromatograms as shown in Fig. 1 (Peak No 1). MALDI-TOF of this peak revealed a mass of 1544.9 ([M + H]^+^) corresponding to the mass of de-iodinated ^nat^Lu-PSMA I&T [C_63_H_90_LuN_11_O_23_; monoisotopic mass = 1543.6 (calculated)], whereas the corresponding mass for the main peak due to ^nat^Lu-PSMA I&T was found at 1669.8 [C_63_H_89_LuIN_11_O_23_; monoisotopic mass = 1669.5 (calculated)]. HPLC sample chromatograms and mass spectrometry can be found in the Additional file 1: Figs. S2 and S3.

### TLC

For TLC system 1 specificity was tested as separation of [^177^Lu]Lu-PSMA I&T (acceptance criteria R_f_ < 0.3) from potential impurities [^177^Lu]Lu-chloride, [^177^Lu]Lu-colloid, and [^177^Lu]Lu-DTPA (acceptance criteria R_f_ > 0.6). In TLC system 1 [^177^Lu]Lu-chloride, [^177^Lu]Lu-colloid, and [^177^Lu]Lu-DTPA migrated with the solvent front with R_f_ values > 0.6, whereas the test solution remained at the origin (R_f_ < 0.2). In TLC system 2 [^177^Lu]Lu-chloride and [^177^Lu]Lu-colloid showed an R_f_ value < 0.4 without significant migration but especially [^177^Lu]Lu-chloride showing pronounced tailing. [^177^Lu]Lu-DTPA and the test solution migrated with the solvent front with R_f_ values > 0.6. Representative radiochromatograms are shown in the Additional file 1: Fig. S4.

### Batch data

In Table 2 results from 8 different batches are summarized. Mean radiochemical purity by HPLC was 96.9 ± 0.5%, analysed by method A; with method B the value was slightly lower with a mean of 96.3 ± 0.9%. This could be explained by the fact that method B was always used after the analysis by method A and indicates some reduction in RCP due to analysing the sample later (up to > 2 h). The amount of PSMA I&T and related substances in these batches resulted in a mean of 98.3 ± 17.3 µg. Mean recovery on both columns was practically quantitative with mean values above 99.9%. TLC resulted in mean radiochemical impurities of less than 0.1% for both TLC systems.

**Table 2:** Batch data (n = 8)

Batch No	RCP HPLC Method A [%]	RCP HPLC Method B [%]	Recovery HPLC Method A [%]	Recovery HPLC Method B [%]	PSMA I&T & related substances [µg/V] (method A)*	TLC System1 Impurity [%]	TLC System2 Impurity [%]
1	96.4	96.4	101.7	101.3	94	0.02	0.14
2	96.6	95.3	100.8	99.3	84	0.03	0.04
3	97.3	95.8	100.8	99.1	119	0.02	0.03
4	97.1	96.8	99.6	98.9	103	0.01	0.03
5	96.3	95.2	98.1	102.5	79	0.10	0.08
6	97.7	97.8	101.1	n.d	n.d	0.01	0.02
7	96.5	96.3	98.7	98.9	87	0.08	n.d
8	97.0	96.8	99.1	99.9	123	0.01	0.03
**Mean**	**96.88**	**96.31**	**99.99**	**99.96**	**98.3**	**0.04**	**0.05**
**Sd**	**0.49**	**0.86**	**1.30**	**1.40**	**17.3**	**0.04**	**0.04**

*V considered as 8 GBq

### Stability study

**Fig. 2:**
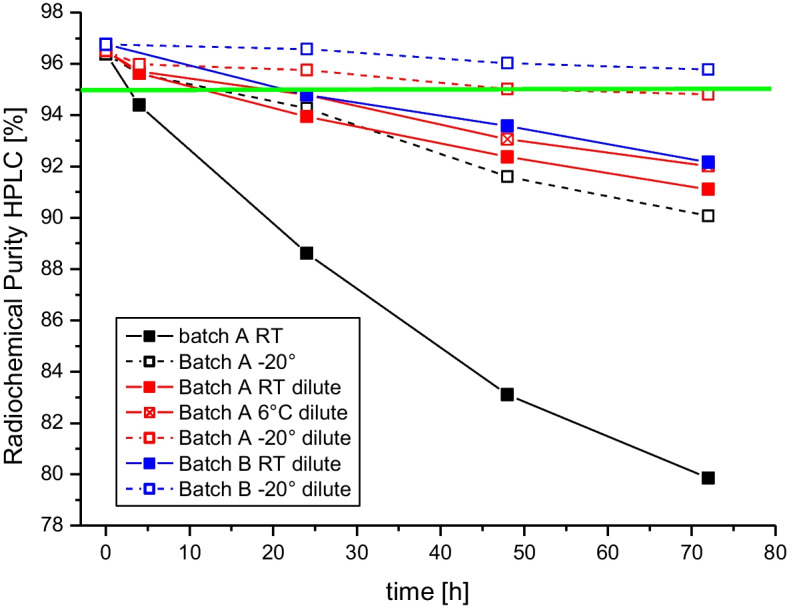
Stability data for 2 batches of [^177^Lu]Lu-PSMA I&T shown as radiochemical purity as determined by HPLC with different storage conditions and different dilutions. Green line indicates the limit of 95%

Stability studies using HPLC revealed a rapid decrease in RCP, whereas with TLC analysis no relevant increase of impurities was detected (impurities < 0.2% in all samples). Samples stored undiluted at room temperature showed RCP below 95% already 4 h after preparation. When the final product sample was diluted with double the volume of ascorbic acid solution (30 mg/mL) degradation was slowed down dramatically, even after 48 h RCP was above 95% when stored at −20 °C. A summary of HPLC results is shown in Fig. 2.

## Discussion

Today, [^177^Lu]Lu-PSMA I&T is widely used for treating advanced prostate cancer and prepared in-house in many centres worldwide on a routine basis. Quality of such a radiopharmaceutical, in particular radiochemical purity, is of utmost importance, especially considering the therapeutic application. Several groups have published methods to determine the quality of this preparation (Orhon et al. 2022; Weineisen et al. 2014; Iorio et al. 2022; Hooijman et al. 2022; Vyas et al. 2022; Aalbersberg et al. 2022), with a focus on RCP testing. However, the quality is not only related to the RCP alone, HPLC analysis allows obtaining confirmation of identity of the radiopharmaceutical and, typically in the form of a limit test, also to determine the amount of precursor and related substances, which should be limited in relation to toxicity concerns. This is well described in several monographs of the European Pharmacopoeia, in particular those on the PSMA ligands [^68^ Ga]Ga-PSMA-11 (European Pharmacopoeia 2022) and [^18^F]F-PSMA-1007 (European Pharmacopoeia 2021), using the UV detector of the HPLC system. By comparing the radioactive peak with the corresponding UV peak of a cold standard, also identity can be assured at the same time. However, only one of the above mentioned publications (Iorio et al. 2022) report on the use of the UV detector in their analysis of [^177^Lu]Lu-PSMA I&T. Identity can in this way only be assured if the analytical system shows appropriate resolution with structurally related substances. Such a resolution is also required to ensure separation of potential impurities, in particular for radiopharmaceutical preparations, where for radioactive impurities no standards are available and often impurities are not identified. Therefore, a system suitability test is described to prove sufficient resolution and specificity. In case of [^68^Ga]Ga-PSMA-11 this is achieved by providing the resolution between the isomers of the complex (European Pharmacopoeia 2022), whereas in the.

[^68^Ga]Ga-Edotreotide monograph the resolution between the precursor Edotreotide and its metal complex ^nat^Ga-Edotreotide is described and the resolution using a mixture of separate standard solutions of these compounds is defined (European Pharmacopoeia 2021). For [^177^Lu]Lu-PSMA I&T none of the reports describe such a test, except for (Orhon et al. 2022), where they use the resolution of separating unbound (“free”) ^177^Lu from [^177^Lu]Lu-PSMA I&T as system suitability test. However, this approach does not meet the requirement of separating structurally related compounds. When establishing the quality control of [^177^Lu]Lu-PSMA I&T in our laboratory we also started with testing a HPLC method based on 0.1% TFA water/ACN mixtures. We soon identified the problem of highly unsymmetric peaks with pronounced tailing for [^177^Lu]Lu-PSMA I&T in the radiometric channel and calculation of sample recovery resulted in values partially below 90%. We did not consider spiking the sample, as it is proposed by Aalbersberg et al. (2022), because this would not allow to quantify the precursor in test samples for a foreseen limit test.

When switching the solvent to phosphate buffer, similar to the solvent described for the analysis of [^18^F]F-PSMA-1007 (European Pharmacopoeia 2021), we found practically quantitative sample recovery and highly symmetric peaks in the radiochromatogram. Optimizing the gradient conditions also allowed a baseline separation of ^nat^Lu-PSMA I&T from the precursor (“free ligand”) PSMA I&T, indicating suitable resolution and specificity of the system. For detection of the precursor and standard solutions we decided to use 200 nm wavelength, where a maximum absorption of PSMA I&T can be found and which provided clearly superior signal to noise ratio as compared to detection at 280 nm (where another maximum can be found, see Additional file 1: Fig. S5). Under the described analytical conditions an LOQ for PSMA I&T of 1 µg/mL was found. This was more than sufficient for our formulations where a minimum concentration of 10 µg/mL was achieved. Considering that for radiolabelling of one patient dose more than 100 µg PSMA I&T are typically applied (Weineisen et al. 2015; Heck et al. 2019; Vyas et al. 2022; Kletting et al. 2016;  Hartrampf et al. 2022), this ensures sufficient sensitivity even when diluted formulations are applied. When analysing routine batches several peaks could be detected in UV, the earliest corresponding in retention time with PSMA I&T, another major peak at the retention time of ^nat^Lu-PSMA I&T, other peaks were smaller and could not be identified, but can be explained being complexes of PSMA I&T with other metals. For quantification of the total amount of PSMA I&T, the sum of the peaks was used for calculation, assuming no relevant differences in absorption of the metal complexes, which has been shown for DOTATATE (Mu et al. 2013), therefore also the term “PSMA I&T and related substances” is used for the test in analogy to Ph. Eur. monographs, e.g. in (European Pharmacopoeia 2022; Pharmacopoeia 2482). In the radiochromatograms of routine samples, several radioactive impurities could be separated, with two peaks eluting after the main peak. These impurities remained stable at low levels of about 1% and therefore might be related to impurities in the precursor. On the other hand, two main radiochemical impurity peaks were eluting before the main peak and one of these increased over time from initial low levels of about 2–2.5%. These, therefore, will be a result from radiolytical processes, that are also known from other similar radiopharmaceuticals, e.g. for somatostatin analogs (Mu et al. 2013),﻿ where tryptophane was identified as the source of modification or minigastrins, where methionine is prone to oxidation (Pawlak et al. 2016). In the case of PSMA I&T it was striking that radiolysis was more pronounced as compared to PSMA-617 radiolabelled under the same conditions. These results are in accordance with findings of Hooijman (Hooijman et al. 2022), who observed as well an increasing side peak over time and eluting before the main peak. Whereas they report a difference in retention time of less than 1 min without baseline separation, in our case this was more than 4 min, supporting the improved high resolution and specificity of our method.

We tried to identify the main radiochemical impurity (Peak 1 in sample chromatograms in Fig. 1). Collection of the peak in decayed samples were inconclusive and not corresponding to any potential degradation product. To access sufficient amounts of compound for mass spectrometry examination, we tried to mimic radiolysis by exposing ^nat^Lu-PSMA I&T to an energy dose of 500 Gy by external radiation of a ^137^Cs-source. The dose of 500 Gy was selected as it corresponds to the radiation dose after about 3–4 h in a sample with a concentration of 2 GBq/mL of Lu-177. Under this condition a UV-peak was detected on HPLC corresponding in retention time to the main radiochemical impurity, and with a mass corresponding to de-iodinated D-iodo-tyrosine building block in ^nat^Lu-PSMA I&T. Therefore radiolysis induced de-iodination is suspected to be the major cause for the instability of [^177^Lu]Lu-PSMA I&T. Data on the in vivo behaviour of this impurity is not available at present. However, we believe it is scientifically interesting to investigate the influence on PSMA binding properties and pharmacokinetics, and hence gain more insight into the consequences of radiolysis.

We initially established the method with a C-12 column (Phenomenex Jupiter Proteo), which met all defined acceptance criteria within our analytical validation in accordance with EANM guidelines (Gillings et al. 2020) and is used for routine analysis. As this type of column is rather uncommon, we also tested the method with a conventional C-18 column. With a slight variation in ACN content, validation provided very comparable results as well, meeting our acceptance criteria and indicating the applicability also when using typical C-18 columns.

Besides of HPLC, for radiometallated radiopharmaceuticals usually a combination with TLC is employed to ensure that any radioactive impurity being retained on the column might not be overlooked. We did not observe such retention in our test samples, as recovery of the HPLC column was practically quantitative. Still we evaluated a TLC method for RCP testing of [^177^Lu]Lu-PSMA I&T. For ^68^ Ga-radiopharmaceuticals, the European Pharmacopoeia describes a test using ITLC-SG with ammonium acetate/methanol mixture as mobile phase. In this system “free” ^68^Ga, either in the hydrolysed form as a colloid or dissolved as an ion, remains at the origin and the ^68^Ga-radiopharmaceutical migrates with the solvent front. Applying this system to [^177^Lu]Lu-PSMA I&T similar results are obtained, as already described by (Orhon et al. 2022). However, it should be considered that it is frequent practice to add DTPA into the final formulation of ^177^Lu-radiopharmaceuticals. [^177^Lu]Lu-DTPA, however, moves with the solvent front and migrates with [^177^Lu]Lu-PSMA I&T, when using ammonium acetate/methanol mixture as mobile phase. We therefore also tested a method using 0.1 M citrate buffer of pH 5 as mobile phase and ITLC-SG as stationary phase. In this system [^177^Lu]Lu-DTPA, [^177^Lu]Lu-chloride and [^177^Lu]Lu-colloids migrate with the solvent front and can be separated from [^177^Lu]Lu-PSMA I&T and used for the determination of “free” ^177^Lu.

## Conclusion

The described HPLC method for quality control of [^177^Lu]Lu-PSMA I&T without the addition of trifluoroacetic acid shows excellent analytical results, which are in line with current Pharmacopoeial standards, while also allowing quantification of PSMA I&T and related substances in the preparation solution. Our validation data confirms the viability of the method on both C-12 and more commonly available C-18 columns. Problems with recovery and tailing as reported previously are overcome by this method. In combination with TLC it provides a reliable tool for quality control of [^177^Lu]Lu-PSMA I&T. We were also able to highlight the importance of adding ascorbic acid to the preparation solution, as [^177^Lu]Lu-PSMA I&T otherwise quickly undergoes degradation, especially if stored at room temperature.

## Supplementary Information

**Additional file ﻿1**. Supplementary information including additional analytical validation data, sample chromatograms and reference Mass- and UV-spectra.

## Data Availability

Supporting information is provided containing additional validation data, results from identification of the radiochemical purity of [^177^Lu]Lu-PSMA I&T, TLC chromatograms and UV–VIS spectrum of PSMA I&T. Additional data are available from the corresponding author upon reasonable request.
