# Supplementary material for: Improved quality control of [177Lu]Lu-PSMA I&T
Source: EJNMMI Radiopharm Chem. 2023 Mar 27;8:7. doi: 10.1186/s41181-023-00191-6 (PMC10043144; doi:10.1186/s41181-023-00191-6)

*Fig S1: Chromatograms for Method B from a different laboratory. From top to bottom: IA and IB (zoom) -radiochromatogram of the test sample of [^177^Lu]Lu-PSMA I&T; II- UV 200 nm chromatogram of the radioactive test sample; III- mixture of PSMA I&T and ^nat^Lu-PSMA I&T UV 200 nm*

[Radio]

I A


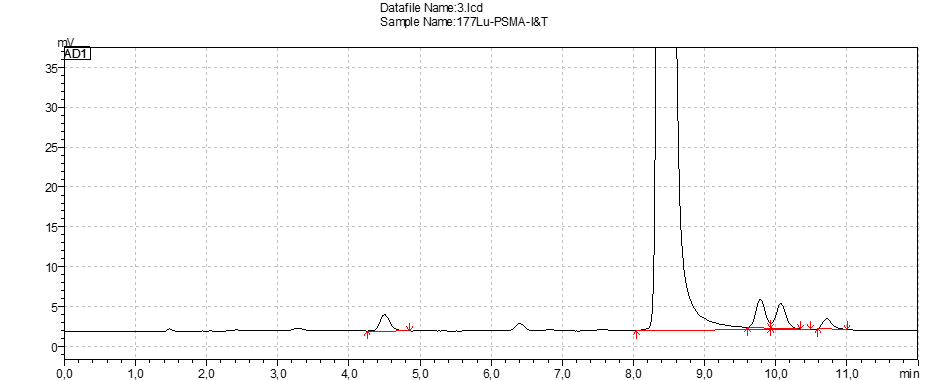


[Radio]

I B

[UV 200nm]

II

[UV 200 nm]

III

*Validation data for Method B from a different laboratory*

| Criteria | Validation parameter | Method | No of repetitions | Aceptance Criteria | Found  Method B  Lab No. 2 |
| --- | --- | --- | --- | --- | --- |
| Identity | Accuracy | Retention time [^177^Lu]Lu-PSMA I&T | 6 | sd (%)<1% | 0.08%  (Rt 8.44min) |
| Identity | Accuracy | Retention time ^nat^Lu-PSMA I&T | 6 | sd (%)<1% | 0.08%  (Rt 8.34min) |
| Identity | Accuracy | Retention time PSMA I&T | 6 | sd (%)<1% | 0.08%  (Rt 7.61min) |
| Identity | Specificity | Retention time [^177^Lu]Lu-PSMA I&T vs ^nat^Lu-PSMA I&T | 6 | deviation < 0.5 min | 0.1 min |
| Radiochemical purity | Specificity | Resolution PSMA I&T vs ^nat^Lu-PSMA I&T | 6 | Resolution >2 | 3.1 |
| Radiochemical purity | Precision | RSD of peak areas of [^177^Lu]Lu-PSMA I&T | 6 | sd (%)≤2% | 1.6% |
| Chemical purity - content | Precision | Sum of the peak areas of PSMA-I&T + Lu-PSMA-I&T | 6 | sd (%)≤2% | 1.8% |

*Fig S2: Chromatograms for PSMA I&T. From top to bottom: I-Radiochromatogram of a test sample of [^177^Lu]Lu-PSMA I&T with a radiochemical purity of about 96% 4 hr post preparation ; II- ^nat^Lu-PSMA I&T UV 220 nm 50µl/mL irradiated with 500Gy; I- ^nat^Lu-PSMA I&T UV 220 nm 50µl/withourt irradiation
Peak labels: 1: main radiochemical impurity; 2: [^177^Lu]Lu-PSMA I&T; 3: impurity of ^nat^Lu-PSMA I&T collected for MALDI-TOF MS; 4: ^nat^Lu-PSMA I&T.*


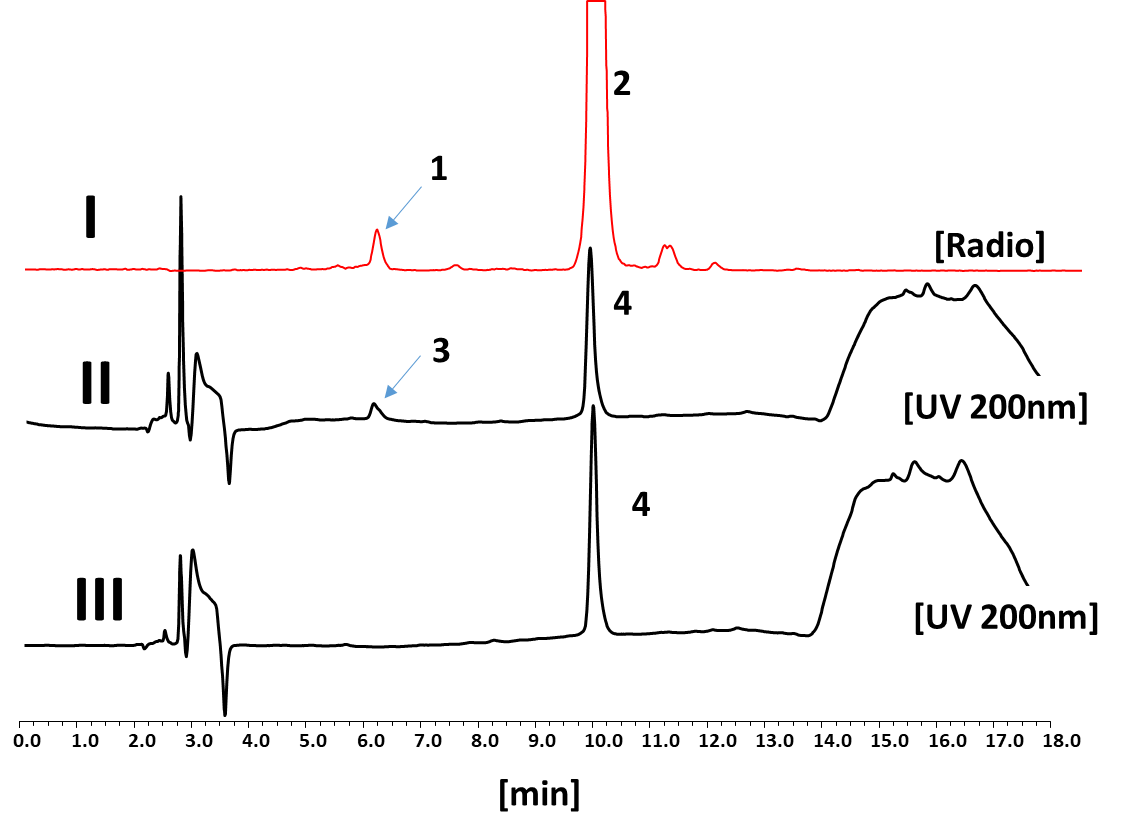


*Fig S3: MALDI-TOF MS of impurity collected after irradiation of ^nat^Lu-PSMA I&T at 500 Gy (Peak 3 according to figure S2).*


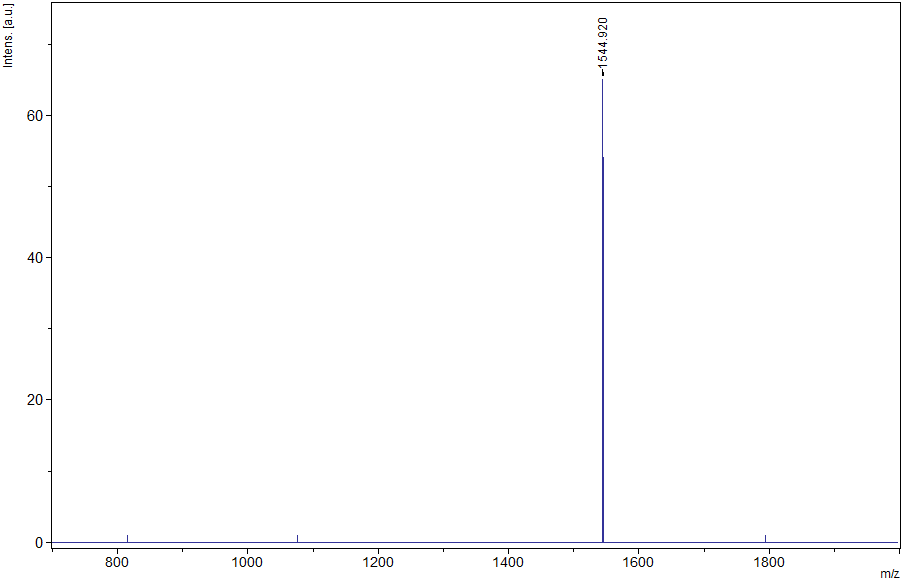


*Figure S4: Representative TLC Chromatograms for [^177^Lu]Lu-Chloride solution, [^177^Lu]Lu-colloid (neutralized [^177^Lu]Lu-Chloride solution), [^177^Lu]Lu-DTPA and the test solution [^177^Lu]Lu-PSMA I&T in TLC system 1 (left) and system 2 (right).*

**[^177^Lu]Lu-Chloride solution**


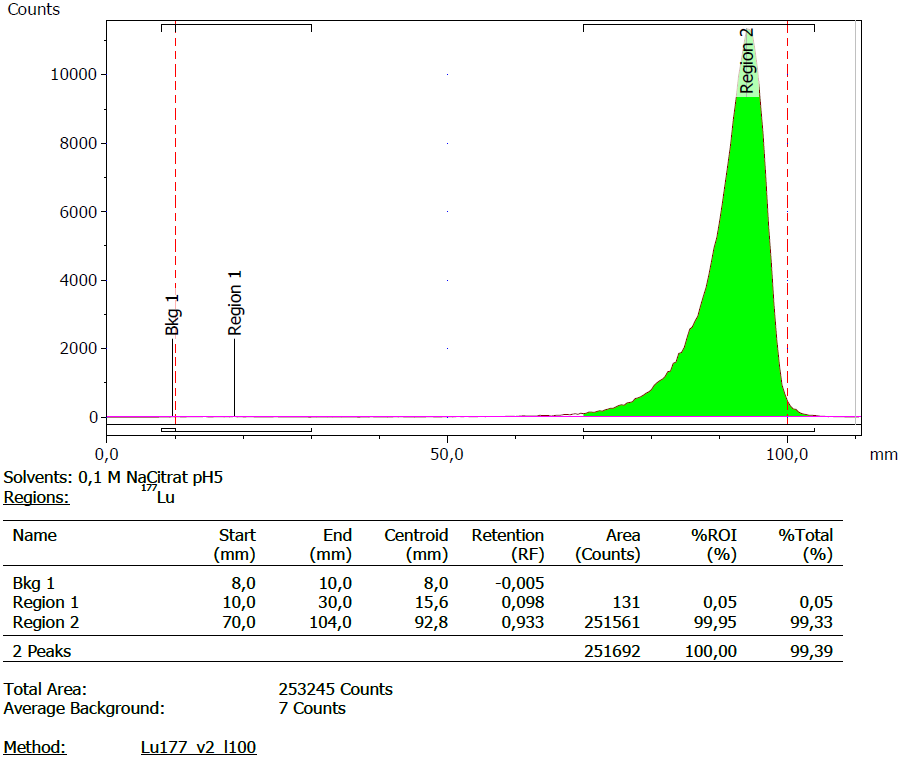

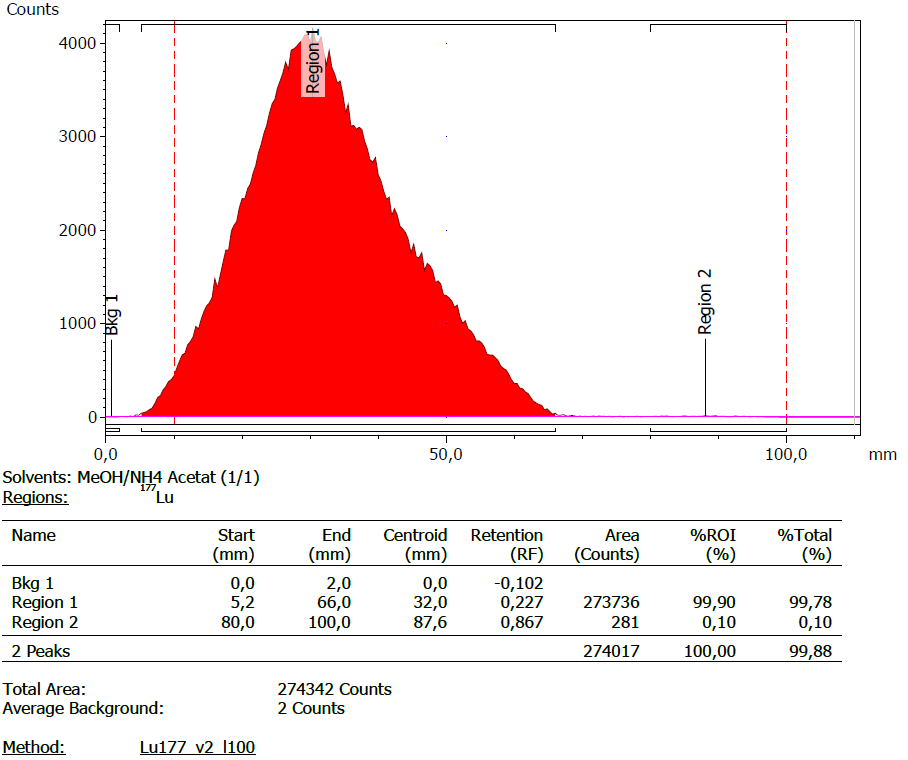


**[^177^Lu]Lu-colloid (neutralized [^177^Lu]Lu-Chloride solution)**


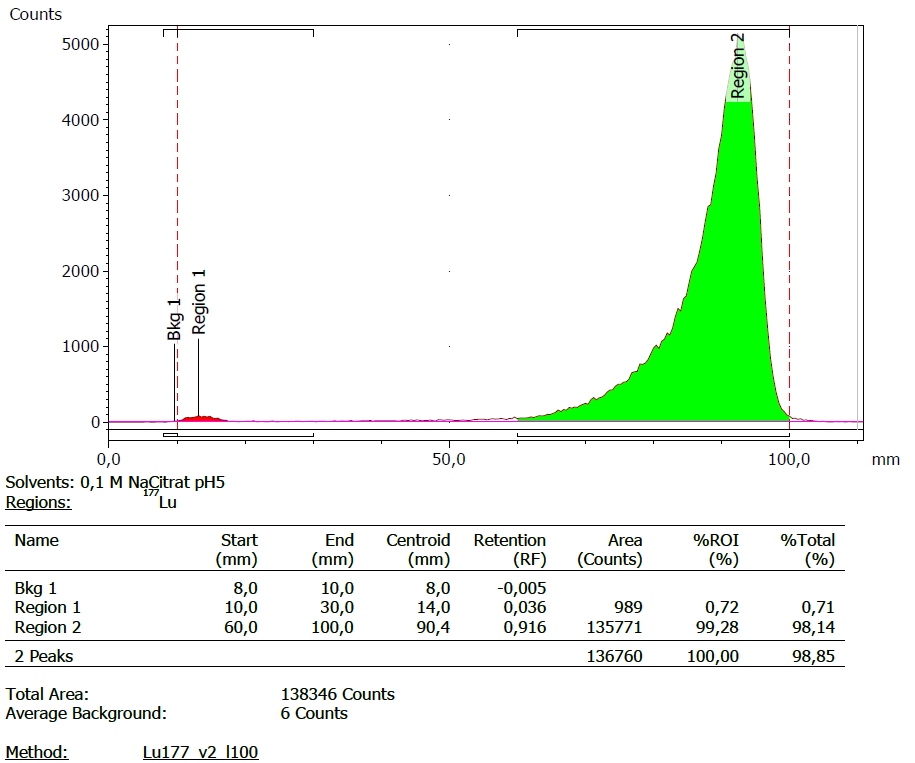

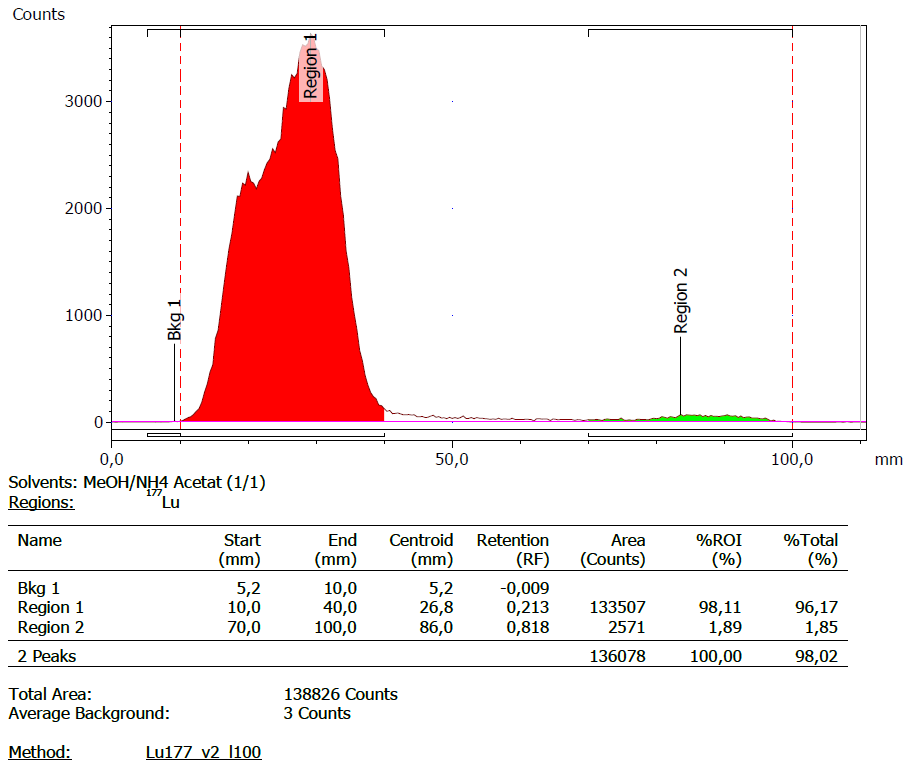


**[^177^Lu]Lu-DTPA**


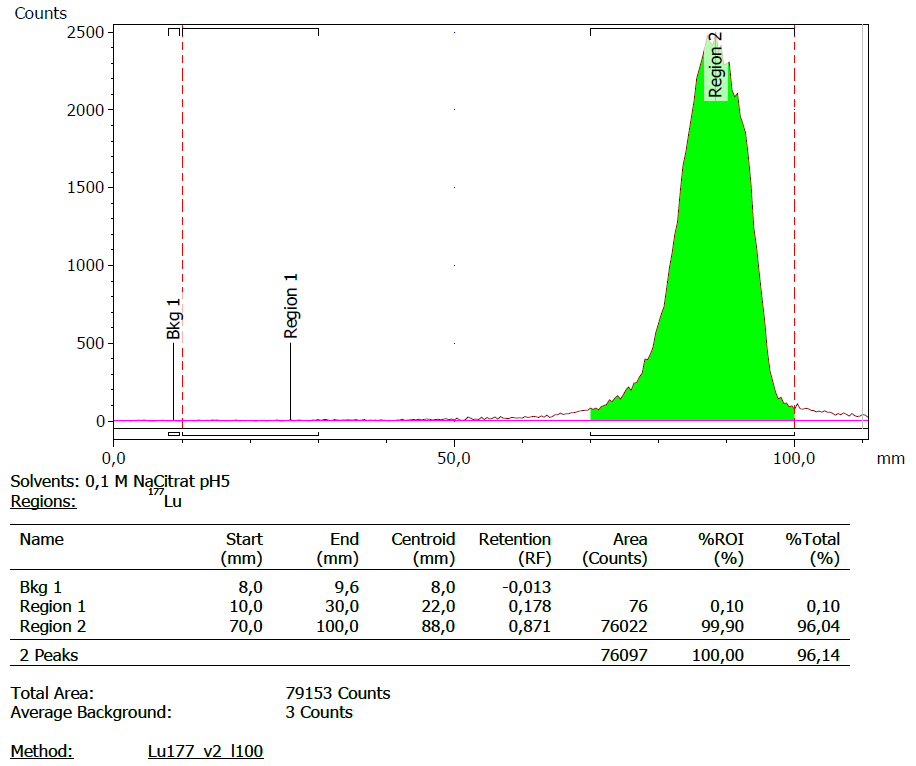

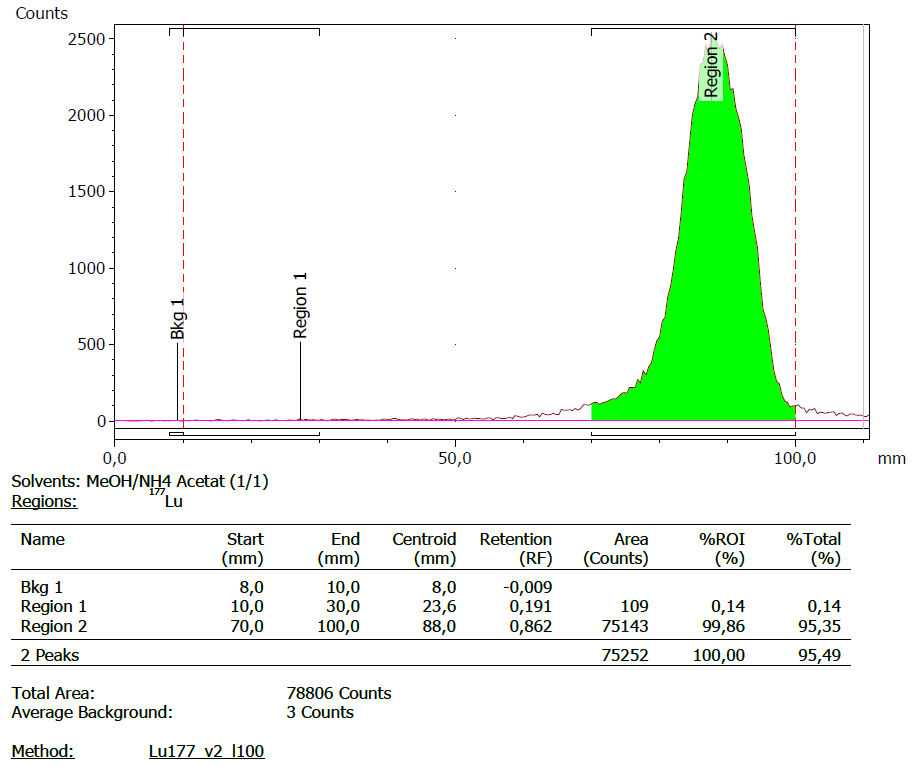


**test solution [^177^Lu]Lu-PSMA I&T**


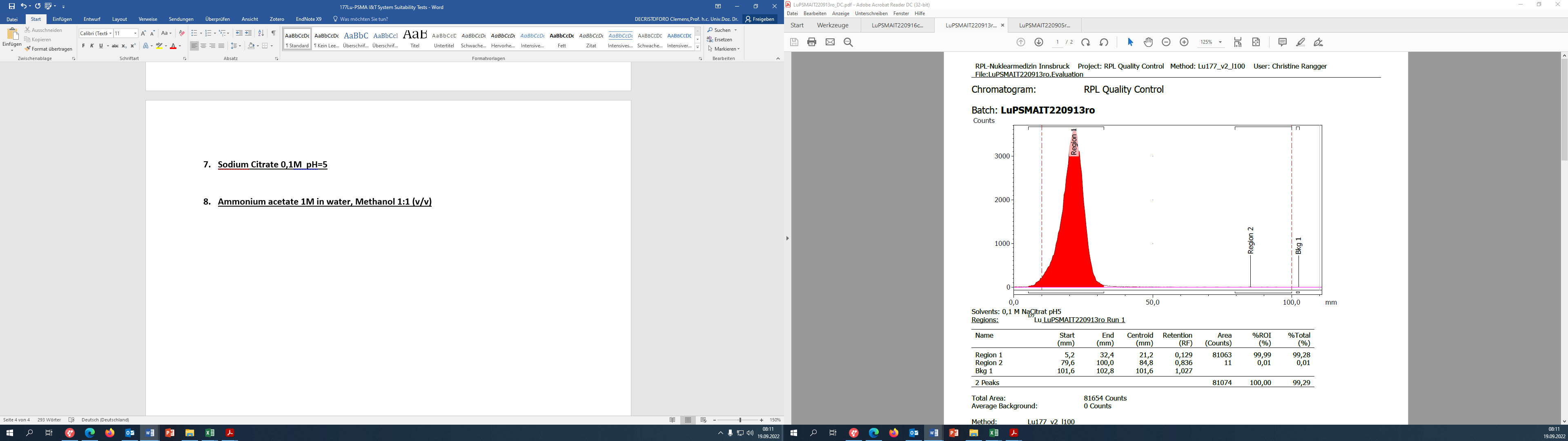

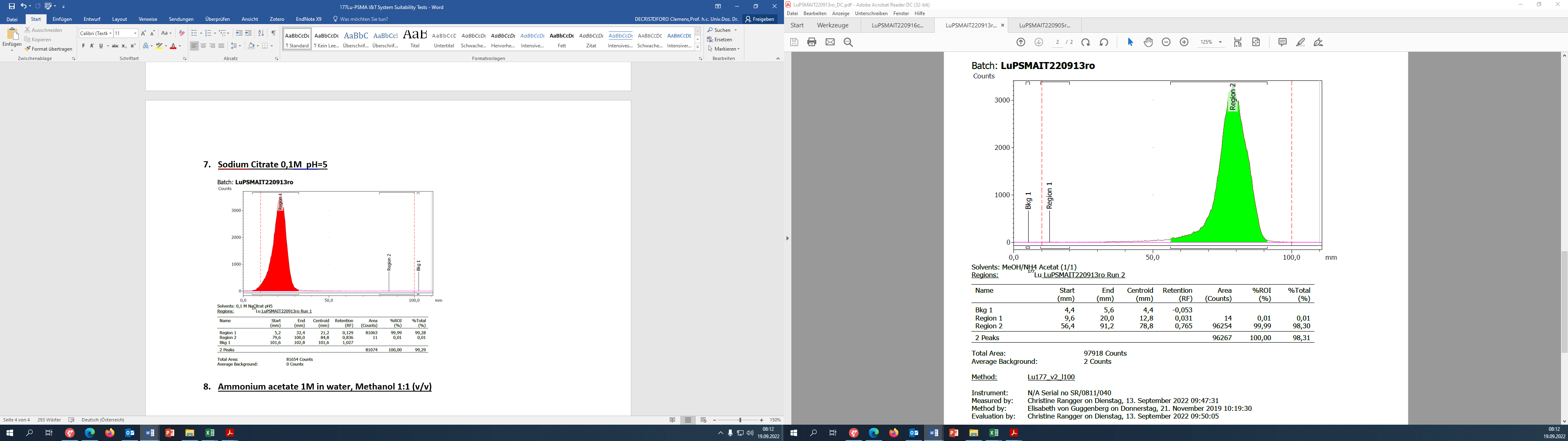


*Figure S5: UV/Vis absorption spectrum of PSMA I&T.*


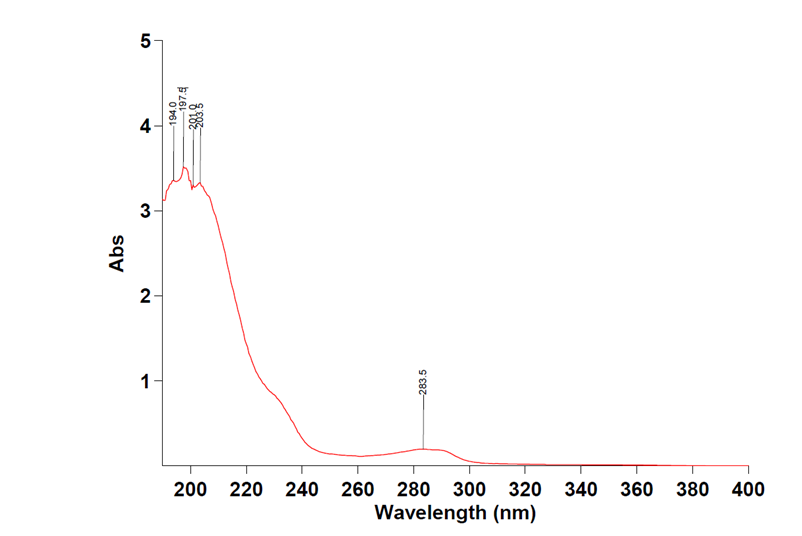

Supplement: Supplementary file 1 — Additional file ﻿1. Supplementary information including additional analytical validation data, sample chromatograms and reference Mass- and UV-spectra. [file 41181_2023_191_MOESM1_ESM.docx]
